# A Simple Nomogram for Predicting Osteoarthritis Severity in Patients with Knee Osteoarthritis

**DOI:** 10.1155/2022/3605369

**Published:** 2022-09-02

**Authors:** Qingzhu Zhang, Yinhui Yao, Jinzhu Wang, Yufeng Chen, Dong Ren, Pengcheng Wang

**Affiliations:** ^1^Orthopedic Trauma Service Center, Third Hospital of Hebei Medical University, Major Laboratory of Orthopedic Biomechanics in Hebei Province, Shijiazhuang, Hebei Province, China; ^2^Department of Orthopedics, The Affiliated Hospital of Chengde Medical University, Chengde, Hebei Province, China; ^3^Department of Pharmacy, The Affiliated Hospital of Chengde Medical University, Chengde, Hebei Province, China

## Abstract

**Objective:**

To explore the influencing factors of knee osteoarthritis (KOA) severity and establish a KOA nomogram model.

**Methods:**

Inpatient data collected in the Department of Joint Surgery, Chengde Medical University Affiliated Hospital from January 2020 to January 2022 were used as the training cohort. Patients with knee osteoarthritis who were admitted to the Third Hospital of Hebei Medical University from February 2022 to May 2022 were taken as the external validation group of the model. In the training group, the least absolute shrinkage and selection operator (LASSO) method was used to screen the factors of KOA severity to determine the best prediction index. Then, after combining the significant factors from the LASSO and multivariate logistic regressions, a prediction model was established. All potential prediction factors were included in the KOA severity prediction model, and the corresponding nomogram was drawn. The consistency index (C-index), area under the receiver operating characteristic (ROC) curve (AUC), GiViTi calibration band, net classification improvement (NRI) index, and integrated discrimination improvement (IDI) index evaluation of a model predicted KOA severity. Decision curve analysis (DCA) and clinical influence curves were used to study the model's potential clinical value. The validation group also used the above evaluation indexes to measure the diagnostic efficiency of the model. Spearman correlation was used to investigate the relationship between nomogram-related markers and osteoarthritis severity.

**Results:**

The total sample included 572 patients with knee osteoarthritis, including 400 patients in the training cohort and 172 patients in the validation cohort. The nomogram's predictive factors were age, pulse, absolute value of lymphocytes, mean corpuscular haemoglobin concentration (MCHC), and blood urea nitrogen (BUN). The C-index and AUC of the model were 0.802. The GiViTi calibration band (*P* = 0.065), NRI (0.091), and IDI (0.033) showed that the modified model can distinguish between severe KOA and nonsevere KOA. DCA showed that the KOA severity nomogram has clinical application value with threshold probabilities between 0.01 and 0.78. The external verification results also show the stability and diagnosis of the model. Age, pulse, MCHC, and BUN are correlated with osteoarthritis severity.

**Conclusions:**

A nomogram model for predicting KOA severity was established for the first time that can visually identify patients with severe KOA and is novel for indirectly evaluating KOA severity by nonimaging means.

## 1. Introduction

Knee osteoarthritis (KOA) is the most common musculoskeletal disease in people over 60 years old, and with the ageing of the population and the prevalence of obesity, the incidence of KOA is on the rise [1, 2]. The incidence of KOA is also on the rise among young people and physically active people [3, 4].

In particular, approximately 10% of people over 55 years old in the world experience KOA pain and incapacitation, making it one of the main causes of disability in the world [5]. According to the data of the third national health and nutrition survey in the United States, the incidence of symptomatic knee osteoarthritis is 12.1% [6]. The prevalence of knee osteoarthritis reported by regional epidemiology in Canada is 10.5%. In addition, China's 2020 research report showed that the number of KOA patients increased from 26.1 million in 1990 to 61.2 million in 2017, and KOA was also the 24th most common cause of disability years in 2017, accounting for 1.08% of all disability years [7].

At present, there is no effective cure for patients with KOA [8]. For a long time, the treatment strategies for KOA have mainly been analgesics and surgery [9–11]. The complications associated with the available treatments pose a huge hidden danger for elderly patients. Nonsteroidal anti-inflammatory drugs are the main drug therapy for osteoarthritis of the knee joint. However, a large number of randomized controlled clinical studies have confirmed that the long-term use of nonsteroidal anti-inflammatory drugs will significantly increase the risk of gastrointestinal bleeding, cardiovascular events, and death [12]. Artificial joint replacement is an important method to treat severe pain and joint deformities in late KOA, but it is not the best choice for patients with a poor economic status or relatively young people because of its high cost and the limited life span of artificial joints. In addition, Beswick et al. reported that nearly 20% of KOA patients still had persistent pain after joint replacement [13]. The proportion of patients having revision surgery within 10 years is as high as 12% [14]. This suggests that it is necessary to explore the factors that affect the severity of knee osteoarthritis to improve the interventions given to patients with early knee osteoarthritis, improve the quality of life of patients, and reduce the social burden.

To date, many studies have focused on the treatment, pathogenesis, and biomarkers of KOA [15, 16]. However, there are few reports that have indirectly evaluated the severity of KOA by nonimaging methods [17–21]. Therefore, by analysing the related data of inpatients in the Department of Joint Surgery, Chengde Medical University Affiliated Hospital, this study investigated the influencing factors of KOA severity, thus establishing a nomogram model. It is hoped that the nomogram can provide a more reliable and accurate visual prediction model. At the same time, the data of inpatients in the Department of Joint Surgery of Third Hospital of Hebei Medical University were used to verify the nomogram model externally.

## 2. Patients and Methods

### 2.1. Data Source

The training cohort retrospectively collected data from a total of 642 patients who were initially diagnosed with KOA in the Department of Joint Surgery, Chengde Medical University Affiliated Hospital from January 2020 to January 2022. A total of 242 patients were excluded due to the lack of clinical data (*n* = 108), combined with osteoarthritis in other joints (*n* = 67), knee replacement, osteotomy and internal fixation for KOA, and knee fracture (*n* = 32), active malignancy (*n* = 10), renal or liver failure (*n* = 10), rheumatic disease (*n* = 9), and active infection (*n* = 6). Finally, the clinical information of 400 KOA patients was collected.

In addition, we selected 256 patients with knee osteoarthritis treated in the Department of Joint Surgery of Third Hospital of Hebei Medical University from February 2022 to May 2022 as the validation cohort. A total of 84 patients were excluded for the following reasons: lack of clinical data (*n* = 42), other joint osteoarthritis (*n* = 10), knee replacement, osteotomy and internal fixation for KOA, and knee fractures (*n* = 12), active malignant tumour (*n* = 3), renal or liver failure (*n* = 5), rheumatic diseases (*n* = 6), and active infection (*n* = 6). Finally, the clinical information of 172 KOA patients was collected.

### 2.2. Data Collection

All clinical information collected in this study was obtained from the examination information of the patients when they were admitted to the hospital. Clinical information of patients included two parts: demographic characteristics and blood laboratory data. Demographic characteristics included the following: sex, age, height, weight, physical illnesses, temperature, pulse, breathing rate, blood pressure, and Kellgren-Lawrence (KL) grade. Blood laboratory data contains a lot of information as follows: C-reactive protein, white blood cell count, red blood cell count, haemoglobin, haematocrit, platelet count, neutrophil ratio, lymphocyte percentage, monocyte percentage, percentage of eosinophils, percentage of basophils, absolute value of neutrophils, absolute value of lymphocytes, absolute value of monocytes, absolute value of eosinophils, absolute value of basophils, average volume of red blood cells, average haemoglobin content, mean corpuscular haemoglobin concentration (MCHC), coefficient of variation of red blood cell distribution width, red blood cell distribution width -SD value, average volume of platelets, distribution width of platelets, ratio of large platelets, thrombocytocrit, total protein, albumin, total bilirubin, prealbumin, alanine aminotransferase, aspartate aminotransferase, gamma glutamyltransferase, direct bilirubin, alkaline phosphatase, blood glucose, total cholesterol, triglyceride, high-density lipoprotein cholesterol, apolipoprotein A1, apolipoprotein B, low-density lipoprotein cholesterol, potassium, sodium, chlorine, calcium, phosphorus, magnesium, *α*-hydroxybutyrate dehydrogenase, lactic dehydrogenase, creatinine kinase, creatine kinase isoenzyme, blood urea nitrogen (BUN), creatinine, uric acid, bicarbonate, *β*2 microglobulin, homocysteine determination, lipoprotein A, serum cystatin C determination, adenosine deaminase, serum total bile acid, estimated glomerular filtration rate, fibrinogen, prothrombin time, thrombin time, activity, international standardized ratio, activated partial thromboplastin time, fibrinogen degradation products, antithrombin III, erythrocyte sedimentation rate, and blood type.

The KL classification system is often used to classify the severity of osteoarthritis using radiological findings. According to the severity of the imaging changes in the bones and joints and by using the KL classification system, KOA can be divided into grades 0, 1, 2, 3, and 4. If there is a classification difference between the patient's knees, the most serious grade is the grading result of the patient [22]. In our study, grade 4 KOA patients were classified into the severe group, while the others (grade 1, 2, and 3 KOA patients) were classified into the nonsevere group.

### 2.3. Construction and Estimation of the Nomogram

Least absolute shrinkage and selection operator (LASSO) methods were used to screen the factors influencing the severity of KOA to determine the best predictive index in the training cohort. Then, by combining the factors obtained by the LASSO regression analysis and multivariate logistic regression analysis, the nomogram of the prediction model was established [10]. *P* < 0.05 indicated that the difference was statistically significant. All potential prediction factors were included in the KOA severity prediction model, and the corresponding nomogram was drawn. Harrell's C statistic was used to calculate the consistency index (C-index) to evaluate the discrimination of the nomogram model. The receiver operating characteristic (ROC) curve was used to calculate the area under the curve (AUC) and evaluate the value of the index model in predicting KOA severity [23]. The GiViTi calibration band was also utilized to illustrate the distinguishing ability of the prediction model. Net reclassification improvement (NRI) and comprehensive discrimination improvement (IDI) indexes were calculated to evaluate the predictive power of the model. Decision curve analysis (DCA) and clinical influence curves were used to study the potential clinical value of the model [24–26]. It is convenient to predict patients with severe KOA in clinical practice. In this study, “DynNom” of the R package was used to support the dynamic statistical analysis of the nomogram model [27].

The factors of the nomogram included in the training cohort were evaluated in the validation cohort. The evaluation indicators in the validation cohort also included the following: AUC, C-index, GiViTi calibration band, and DCA.

### 2.4. Statistical Analysis

All data in this study were analysed by the R software (version 4.1.2; https://www.r-project.org/). In this study, the comparison of continuous variables between the two groups is expressed as the mean, standard deviation, and difference. Student's *t*-test was used for normally distributed data, but the Mann–Whitney *U* test was used for nonnormally distributed data. The R package used in the LASSO method is “glmnet.” The AUC, C-index, GiViTi calibration band, and DCA adopted the R packages “pROC,” “Hmisc,” “givitiR,” and “rms,” respectively. The use of NRI and IDI includes the R packages “nricens” and “PredictABEL.” Spearman grade correlation coefficients were calculated to investigate the relationship between nomogram-related markers and osteoarthritis severity by the R software.

## 3. Results

### 3.1. Characteristics of the KOA Patients

The training cohort included 400 patients (110 males and 290 females) with an average age of 64 (58, 69) years. According to the KL grading system, the patients were divided into two groups: the KL 1-3 KOA group (206 cases) and the KL 4 KOA group (194 cases). The demographic characteristics, blood laboratory results, and knee osteoarthritis grouping of the two groups (severe group vs. nonsevere group) are shown in Table 1. In the comparison between the severe group and the nonsevere group, the variables with significant differences (*P* < 0.05) included age, physical illnesses, pulse, systolic pressure, red blood cell count, haemoglobin, haematocrit, lymphocyte percentage, absolute value of lymphocytes, average haemoglobin content, mean corpuscular haemoglobin concentration (MCHC), coefficient of the variation of red blood cell distribution width, red blood cell distribution width (SD value), albumin, total bilirubin, alkaline phosphatase, potassium, sodium, *α*-hydroxybutyrate dehydrogenase, lactic dehydrogenase, blood urea nitrogen (BUN), *β*2 microglobulin, homocysteine determination, serum cystatin C, glomerular filtration rate, and erythrocyte sedimentation rate.

There were 172 KOA patients (43 males and 129 females) in the validation cohort, with an average age of 62.41 ± 6.36 years (Table S1). The patients can be divided into two groups by the same grading method: the KL 1-3 KOA group and the KL 4 KOA group. Because the blood laboratory results of different hospitals contain different items, the validation cohort lacks the red blood cell distribution width (SD), ratio of large platelets, *β*2 microglobulin, serum cystatin C, adenosine deaminase and estimated glomerular filtration rate. The comparison between the severe group and the nonsevere group in the validation group shows that there are seven variables with the same significant differences as those in the training group: age, pulse, systolic pressure, average haemoglobin content, mean corpuscular haemoglobin concentration (MCHC), coefficient of the variation of red blood cell distribution width, and homocysteine determination. The other four variables with significant differences were breathing rate, prealbumin, gamma glutamyltransferase, and fibrinogen degradation products.

### 3.2. Nomogram Variable Screening and Construction

**Figure 1:**
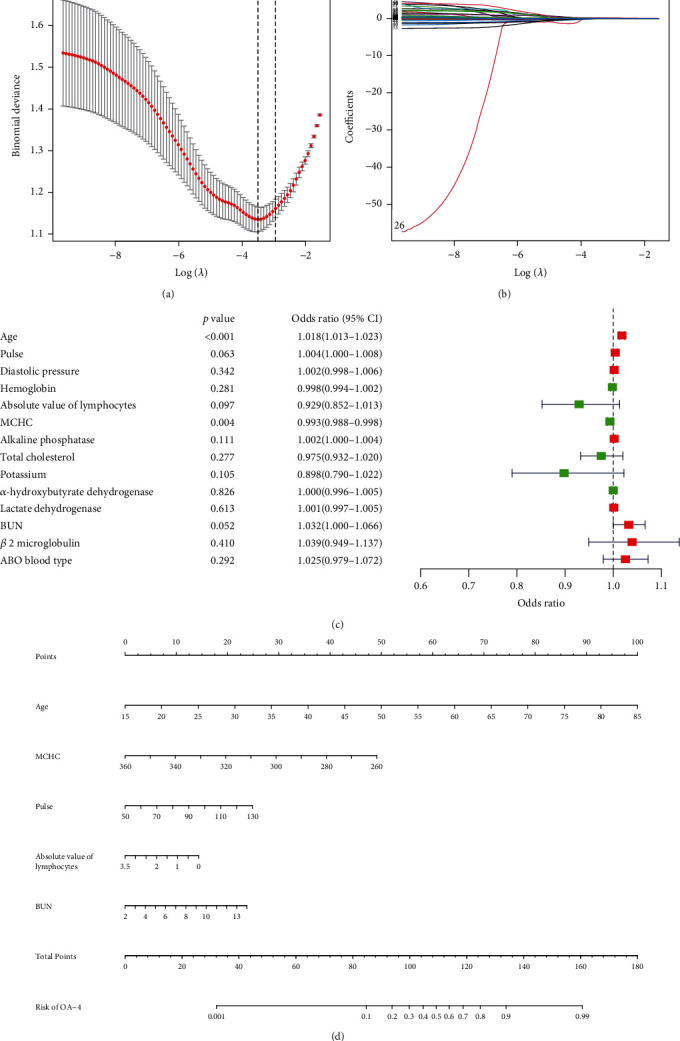
Prediction factors for osteoarthritis severity were selected, and an osteoarthritis severity nomogram was developed in patients with knee osteoarthritis in the training cohort. (a, b) Least absolute shrinkage and selection operator (LASSO) coefficient profiles of the 14 prediction factors. (c) Logistic regression analyses of the 5 prediction factors in patients with knee osteoarthritis. (d) Nomogram prediction of osteoarthritis severity in patients with knee osteoarthritis.

In the LASSO regression analysis of the training cohort, 400 patients had 81 features, which were reduced to 14 potential nonzero coefficient predictors related to KOA. These 14 factors are as follows: age, pulse, diastolic pressure, haemoglobin, absolute value of lymphocytes, MCHC, alkaline phosphatase, total cholesterol, potassium, *α*-hydroxybutyrate dehydrogenase, lactate dehydrogenase, BUN, *β*2 microglobulin, and ABO blood type (Figures 1(a) and 1(b)). As determined by the multivariate logistic regression analysis of the above 14 factors, only the *P* values of age and MCHC were less than 0.05, and the *P* values of pulse, absolute value of lymphocytes, and BUN were less than 0.1 (Figure 1(c)). Finally, the above five factors were included in the nomogram model to predict the severity of KOA (Figure 1(d)). In this study, a dynamic nomogram was used to visually demonstrate the diagnostic performance of these five variables (age, MCHC, pulse, absolute value of lymphocytes, and BUN) for severe KOA (Figure S1).

### 3.3. Evaluation of the Nomogram

**Figure 2:**
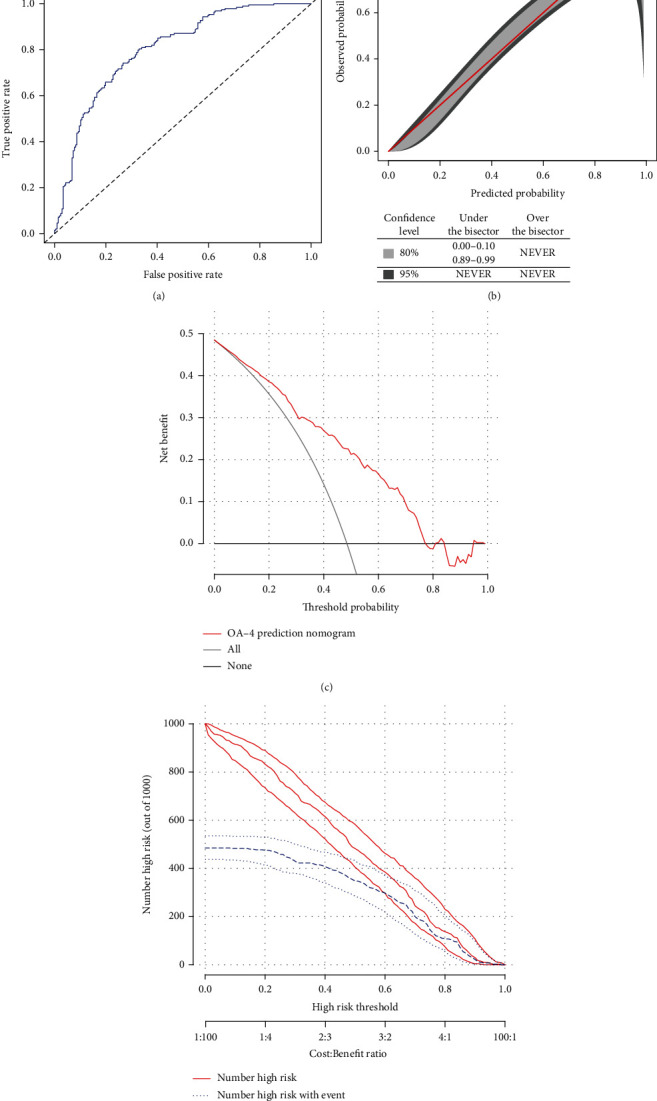
Evaluation of the KOA nomogram and its clinical use in patients with KOA in the training cohort. (a) ROC curve based on the predictive nomogram for osteoarthritis severity. (b) Calibration plots for predicting osteoarthritis severity. (c) Decision curve analysis for the osteoarthritis severity nomogram in patients with knee osteoarthritis. (d) Clinical impact plot for predicting osteoarthritis severity.

The C-index and AUC were 0.802, which indicates that the nomogram has a good degree of discrimination for the severity of KOA (Figure 2(a)). The GiViTi calibration curve (*P* = 0.065) in this study also consistently showed a good nomogram (Figure 2(b)). The changes in the NRI and IDI were used to compare the accuracy between the nomogram model and the two-variable model (the model established by age and MCHC). The NRI and IDI were 0.091 and 0.033, respectively (both *P* < 0.05). In addition, the AUC of the nomogram was higher than that of the two-variable model (0.802 vs. 0.783, *P* < 0.05). These indicators show that the nomogram is more accurate than the two-variable model.

### 3.4. Clinical Use of the Nomogram

This study predicts severe DCA of KOA, as shown in Figures 2(c) and 2(d). The DCA results show that the nomogram that was used to differentiate severe KOA in this study population is more beneficial than all of the patient intervention or nonintervention schemes because it has a threshold probability of 0.01-0.78 (Figure 2(c)). In addition, the clinical impact chart shows that the predicted number of high-risk patients is always greater than the actual number of noncompliant patients, which seems to be accompanied by an acceptable cost–benefit ratio (Figure 2(d)). These results indicate that the nomogram has high clinical application potential for determining the severity of KOA patients.

### 3.5. Validation of the Nomogram

**Figure 3:**
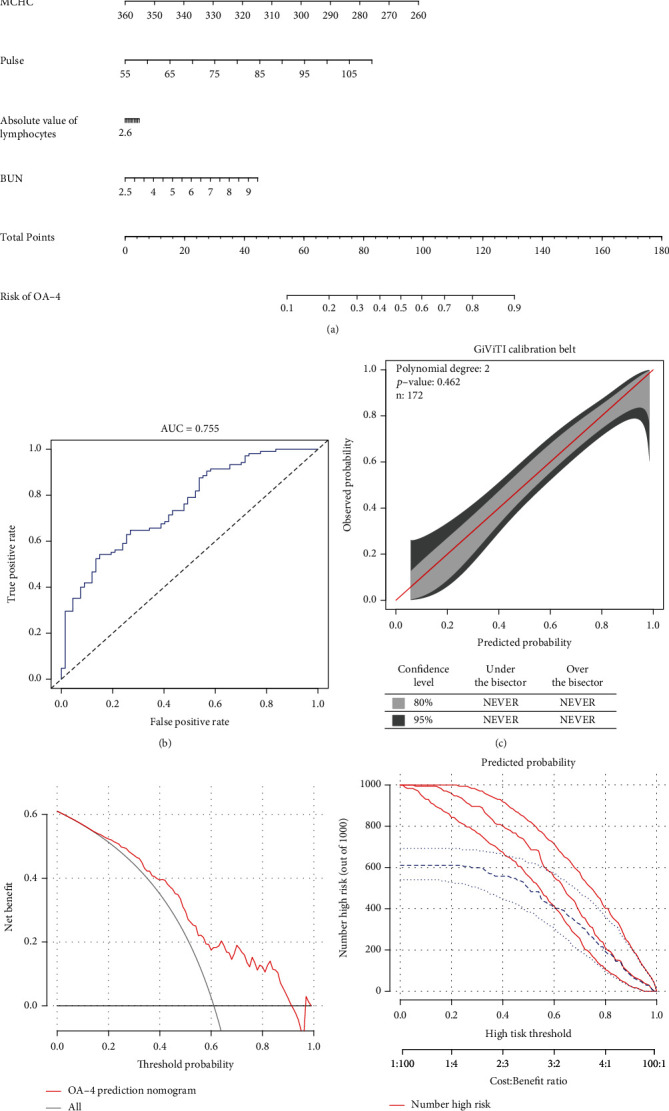
Evaluation of the KOA nomogram and its clinical use in patients with KOA in the validation cohort. (a) Nomogram prediction of osteoarthritis severity in patients with knee osteoarthritis. (b) ROC curve based on the predictive nomogram for osteoarthritis severity. (c) Calibration plots for predicting osteoarthritis severity. (d) Decision curve analysis for the osteoarthritis severity nomogram in patients with knee osteoarthritis. (e) Clinical impact plot for predicting osteoarthritis severity.

The nomogram model in the training cohort included age, MCHC, pulse, absolute value of lymphocytes, and BUN (Figure 3(a)). Then, the same variables as those of the training cohort were used in the validation cohort to construct a diagnosis model for patients with severe KOA, and the nomogram model was evaluated. In the validation cohort, both the C-index and AUC were 0.755 (Figure 3(b)). In addition, the *P* value of the GiViTi calibration curve was 0.462 (Figure 3(c)). These three evaluation indexes all show that the nomogram model has certain value in the diagnosis of patients with severe KOA in the validation cohort. DCA was performed in the validation cohort to estimate the net benefit to patients (Figure 3(d)). DCA showed the obvious net benefits of the nomogram model for almost all threshold probabilities (Figure 3(e)), especially the threshold probabilities between 5 and 91% (Figure 3(d)).

### 3.6. Correlations between Nomogram-Related Markers and Osteoarthritis Severity

**Figure 4:**
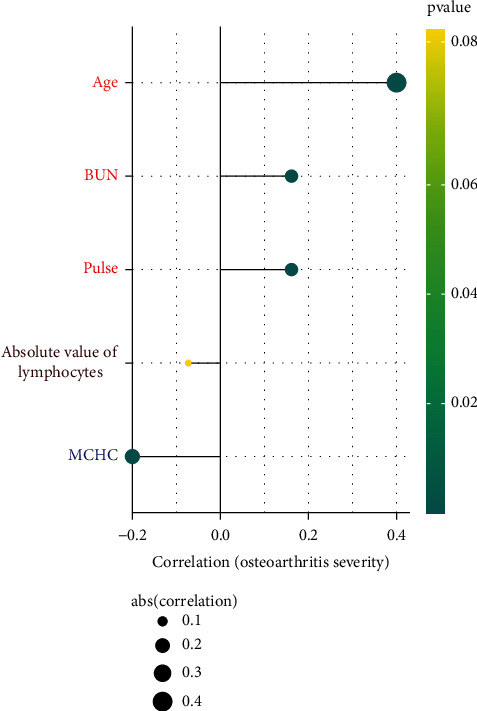
Correlation analysis of nomogram-related markers and osteoarthritis severity.

In both the training cohort and the validation cohort, age, MCHC, pulse, absolute value of lymphocytes, and BUN were well presented as the factors included in the establishment of the nomogram model. Spearman correlation analysis showed that except the absolute value of lymphocytes, other indicators were correlated with the severity of osteoarthritis (Figure 4). Age (*r* = 0.4), pulse (*r* = 0.16), and BUN (*r* = 0.16) were positively related to the osteoarthritis severity. MCHC (*r* = −0.2) is negatively proportional to the osteoarthritis severity.

## 4. Discussion

KOA is a chronic disease occurring in the knee joint caused by the interaction of many factors; it is characterized by articular cartilage degeneration and secondary bone hyperplasia. As the most common joint disease, it is estimated that 302 million people in the world are affected by KOA, and it has become one of the main causes of disability in the elderly [8, 28, 29]. Epidemiological survey data in China show that the prevalence rate of symptomatic KOA in China is 8.1% at present, and frequent knee pain affects the activity and quality of life of up to 25% of adults [30]. The high prevalence and disability rate of KOA have greatly affected the patients' quality of life and social and economic development. During the early stage of KOA, the articular cartilage still has a certain regenerative capacity, but during the late stage of KOA, the articular cartilage may permanently lose its regenerative capacity [31, 32]. According to the diagnosis and treatment of KOA, experts have divided KOA into early, middle stage, and late stages. In the early stage, drug treatment is recommended, but in the middle and late stages, invasive treatments such as repair and joint replacement are recommended [33]. Therefore, early identification of the severity of KOA plays an important role in the treatment and prognosis of KOA.

The nomogram model can visualize the results of logistic regression and can be directly used to predict the individual disease risk, which is easy to popularize and apply in the clinic. Studies at home and abroad have confirmed that nomogram models can be used to predict the prognosis of hepatocellular carcinomas, melanomas of the head and neck, gliomas, young patients with gastric cancer, and the risk of anastomotic leakage after rectal cancer surgery [34–38]. In the field of KOA, the prediction accuracy and clinical value of nomograms have also been confirmed, and nomograms can be used to predict the probability of replacement surgery in the late stage of KOA and the probability of complications after joint replacement [18, 39]. However, there is little literature on the establishment of a nomogram model of KOA severity that is related to the clinical application of X-ray films to evaluate KOA severity. Based on the abovementioned influencing factors of KOA severity, a nomogram model for predicting KOA severity was established for the first time, which realized visual and individualized prediction, helped to formulate strategies to prevent KOA, supplemented the shortcomings of imaging methods in evaluating KOA severity, and proposed a new method for indirect evaluation of KOA severity by nonimaging methods. In clinical work, the nomogram model of this study can be used in primary medical units without access to imaging equipment (for example, community health service stations), in patients who are unwilling to receive radiation, in patients who cannot receive radiation (for example, pregnant women), and in patients who have been bedridden for a long time and have difficulty with X-ray examinations.

A large number of studies have reported the relationship between age and KOA. Jurmain found that the incidence of osteoarthritis increased with age [39]. Calce et al. found that most of the changes in KOA patients can be explained by age [40]. Deng et al. suggested that ageing is the key driving force of osteoarthritis [41]. Zhang et al. reported that osteoarthritis is an age-related arthritis and the main cause of chronic disability in the elderly [42]. This study is consistent with the above conclusions: it was found that age is an independent risk factor for patients with severe KOA. With increasing age, the severity of KOA increased (*r* = 0.4, *P* < 0.001).

There is no literature that directly supports the correlation between pulse and KOA severity. However, a large number of studies have proven that cardiovascular disease (CVD) is closely related to osteoarthritis, and there is a positive correlation [43–46]. Moreover, some studies have pointed out that vascular lesions around joints are one of the pathogeneses of osteoarthritis, and these vascular lesions have been proven to be similar to CVD in pathology and are considered to be a manifestation of systemic metabolic abnormalities [47], which further verifies the close relationship between CVD and osteoarthritis. These considerations make it easier for us to understand the results of this study: pulse is an independent risk factor for patients with severe KOA, and with the acceleration of the pulse, the severity of KOA increases (*r* = 0.16, *P* < 0.001). Output per stroke is an important indicator of cardiac function. The greater the output per stroke, the better the cardiac function. Under the same cardiac output, the faster the pulse is, the smaller the stroke output; the slower the pulse is, the larger the stroke output. However, CVD is positively correlated with osteoarthritis. It has been found that the faster the pulse and the smaller the output per pulse, the worse the heart function and the more severe the osteoarthritis, which could explain the results of our study.

BUN is a nitrogen-containing compound in the plasma and is filtered out from the glomerulus and excreted. When renal insufficiency is decompensated, BUN will increase. Therefore, BUN is used as an index to evaluate glomerular filtration function in clinical work. There is no literature to support that BUN is directly related to KOA. However, the literature has proven that BUN increases with age [48], and age is closely related to KOA [39–42]. These conclusions can fully explain the results of our study; the higher the BUN (*r* = 0.16, *P* < 0.001) is, the heavier the severity of KOA.

Many scholars have found that the absolute value of lymphocytes is inversely related to the severity of KOA [49–52]; that is, the smaller the absolute value of lymphocytes is, the heavier the severity of KOA. Additionally, the larger the absolute value of lymphocytes is, the lighter the severity of KOA. This is consistent with our research results.

Many studies have reported the importance of low MCHC in predicting the prognosis of diseases [53–55], including hepatectomy, chronic obstructive pulmonary disease, and the development of cardiovascular diseases in dialysis patients. However, no literature has proven the relationship between MCHC and KOA. MCHC is defined as the amount of haemoglobin per litre of blood/haematocrit per litre of blood. There is a positive correlation between MCHC and haemoglobin, and it has been reported in the literature that haemoglobin tends to decrease with age [56], so MCHC also tends to decrease with age. Age is closely related to KOA [36–39]. This finding fully explains the results of this study, which showed that with a decrease in MCHC (*r* = −0.2, *P* < 0.001), the severity of KOA increases.

The C-index of KOA severity predicted by the nomogram model in this study was 0.802. The internal verification shows that the KOA severity predicted by this model is in good agreement with the actual KOA severity. The calibration curve further verifies that the model prediction has excellent discrimination and accuracy. In addition to excellent prediction accuracy, this study also confirmed that the nomogram model can effectively predict KOA severity by ROC curve analysis. To avoid data overfitting in the process of building the nomogram model in the training cohort, this study used external data for verification. The AUC performance of the validation cohort was as good as that of the training cohort. There was no significant difference in AUC between the training cohort and the validation cohort (*P* = 0.272). This also further shows that the nomogram model has good discrimination for severe KOA from patients with nonsevere KOA in the validation cohort. By introducing a clinical decision curve and clinical influence curve to investigate the advantages and disadvantages of statistical inference results, the results further confirmed that this model has strong clinical practicability and high benefit in the training cohort and validation cohort.

The limitations of this study are as follows: (1) the sample size is small; (2) the nomogram for predicting KOA severity needs to be further verified by multicentre and large-scale case studies.

## 5. Conclusions

In this study, a nomogram model for predicting KOA severity was established for the first time by combining five influencing factors, including age, pulse, absolute value of lymphocytes, MCHC, and BUN. Individualized prediction of KOA severity can be obtained, and these can help to directly identify patients with severe KOA, help to formulate strategies for preventing KOA, and may open up new ideas for indirectly evaluating KOA severity by nonimaging means.

## Figures and Tables

**Table 1:** Demographics and clinical characteristics of 400 patients with knee osteoarthritis in the training cohort.

Variables	Total (*n* = 400)	KL 1-3 (*n* = 206)	KL 4 (*n* = 194)	*P*
Sex, n (%)				0.19
Female	290 (72)	143 (69)	147 (76)	
Male	110 (28)	63 (31)	47 (24)	
Age, median (Q1, Q3)	64 (58, 69)	60 (54.25, 65)	66 (63, 71)	<0.001
Height, median (Q1, Q3)	160 (158, 167)	162 (158, 168)	160 (158, 165)	0.147
Weight, median (Q1, Q3)	70 (60, 75)	70 (62, 80)	69 (60, 75)	0.061
Physical illnesses, *n* (%)				<0.001
No	152 (38)	96 (47)	56 (29)	
Yes	248 (62)	110 (53)	138 (71)	
Temperature, median (Q1, Q3)	36.4 (36.2, 36.6)	36.3 (36.2, 36.6)	36.4 (36.2, 36.5)	0.635
Pulse, median (Q1, Q3)	80 (74, 88)	80 (72, 87)	82 (74.5, 90)	0.004
Breathing rate, *n* (%)				0.77
16	34 (8)	16 (8)	18 (9)	
18	256 (64)	135 (66)	121 (62)	
20	110 (28)	55 (27)	55 (28)	
Systolic pressure, median (Q1, Q3)	141 (130, 157)	138 (128, 150)	146 (132.25, 160)	0.003
Diastolic pressure, mean ± SD	83.05 ± 11.83	82.71 ± 12.06	83.41 ± 11.61	0.555
C-reactive protein, median (Q1, Q3)	1.68 (0.81, 3.95)	1.68 (0.83, 3.62)	1.74 (0.8, 4.3)	0.462
White blood cell count, median (Q1, Q3)	5.64 (4.75, 6.49)	5.64 (4.79, 6.42)	5.65 (4.72, 6.64)	0.807
Red blood cell count, median (Q1, Q3)	4.33 (4.09, 4.65)	4.39 (4.13, 4.71)	4.24 (3.99, 4.56)	<0.001
Haemoglobin, median (Q1, Q3)	132 (123, 142)	134 (127, 144)	128 (120.25, 138)	<0.001
Haematocrit, median (Q1, Q3)	40 (37.9, 42.73)	40.65 (38.52, 43.18)	39.4 (37.25, 42.08)	<0.001
Platelet count, mean ± SD	225.93 ± 54.4	224.91 ± 52.99	227 ± 55.97	0.702
Neutrophil ratio, mean ± SD	58.04 ± 8.96	57.21 ± 9.15	58.91 ± 8.69	0.057
Lymphocyte percentage, mean ± SD	31.36 ± 7.9	32.29 ± 8.05	30.38 ± 7.63	0.015
Monocyte percentage, median (Q1, Q3)	7.4 (6.4, 8.6)	7.3 (6.4, 8.6)	7.5 (6.5, 8.78)	0.285
Percentage of eosinophils, median (Q1, Q3)	2 (1.2, 3.1)	2.05 (1.2, 3)	2 (1.2, 3.1)	0.937
Percentage of basophils, median (Q1, Q3)	0.5 (0.4, 0.7)	0.5 (0.4, 0.7)	0.5 (0.4, 0.7)	0.337
Absolute value of neutrophils, median (Q1, Q3)	3.16 (2.59, 3.93)	3.1 (2.63, 3.75)	3.34 (2.55, 4.09)	0.184
Absolute value of lymphocytes, median (Q1, Q3)	1.71 (1.38, 2.12)	1.78 (1.43, 2.17)	1.67 (1.35, 2.05)	0.043
Absolute value of monocytes, median (Q1, Q3)	0.42 (0.34, 0.5)	0.41 (0.34, 0.49)	0.42 (0.34, 0.51)	0.236
Absolute value of eosinophils, median (Q1, Q3)	0.11 (0.07, 0.17)	0.11 (0.07, 0.17)	0.11 (0.06, 0.18)	0.845
Absolute value of basophils, median (Q1, Q3)	0.03 (0.02, 0.04)	0.03 (0.02, 0.04)	0.03 (0.02, 0.04)	0.486
Average volume of red blood cells, median (Q1, Q3)	92.8 (89.8, 95.7)	92.5 (90.23, 94.97)	93.05 (89.5, 96)	0.382
Average haemoglobin content, median (Q1, Q3)	30.6 (29.58, 31.6)	30.9 (29.8, 31.8)	30.45 (29.2, 31.4)	0.011
Mean corpuscular haemoglobin concentration(MCHC), median (Q1, Q3)	329 (322, 336)	332 (324.25, 338)	325.5 (320, 333)	<0.001
Coefficient of the variation of red blood cell distribution width, median (Q1, Q3)	12.6 (12.1, 13.1)	12.5 (12, 13)	12.6 (12.12, 13.2)	0.003
Red blood cell distribution width -SD value, median (Q1, Q3)	42.9 (41, 44.9)	42.2 (40.8, 44.4)	43.45 (41.73, 45.5)	<0.001
Average volume of platelets, median (Q1, Q3)	10.3 (9.7, 11)	10.3 (9.6, 11)	10.3 (9.9, 11)	0.258
Distribution width of platelets, median (Q1, Q3)	11.7 (10.6, 13.4)	11.7 (10.5, 13.4)	11.7 (10.8, 13.35)	0.537
Ratio of large platelets, median (Q1, Q3)	27.25 (22.4, 33.32)	27.25 (21.83, 33.27)	27.2 (23.58, 33.25)	0.377
Thrombocytocrit, median (Q1, Q3)	0.23 (0.2, 0.26)	0.23 (0.2, 0.26)	0.24 (0.2, 0.27)	0.505
Total protein, median (Q1, Q3)	68.1 (64.65, 71.6)	67.95 (65.2, 71.75)	68.15 (63.9, 71.38)	0.53
Albumin, median (Q1, Q3)	38.8 (37, 40.73)	39.2 (37.4, 41.08)	38.4 (36.6, 40.48)	0.006
Total bilirubin, median (Q1, Q3)	11.77 (9.44, 14.66)	12.41 (9.72, 14.98)	11.22 (9.13, 14)	0.034
Prealbumin, median (Q1, Q3)	250.45 (213.75, 287.82)	254.1 (216.4, 291.65)	241.7 (208.62, 284.35)	0.093
Alanine aminotransferase, median (Q1, Q3)	15 (11.2, 21.38)	15.4 (11.25, 21.28)	14.1 (11.12, 21.48)	0.244
Aspartate aminotransferase, median (Q1, Q3)	19.1 (16.28, 23.4)	19.35 (16.83, 23.4)	18.9 (16.1, 23.37)	0.542
Gamma glutamyltransferase, median (Q1, Q3)	22.05 (15.9, 34.45)	21.75 (15.72, 33.6)	22.55 (16.52, 35.55)	0.496
Direct bilirubin, median (Q1, Q3)	3.3 (2.5, 4.2)	3.5 (2.5, 4.38)	3.2 (2.4, 4)	0.151
Alkaline phosphatase, median (Q1, Q3)	82.2 (69.7, 98.1)	76.85 (67.53, 91.95)	86.55 (72.9, 103.6)	<0.001
Blood glucose, median (Q1, Q3)	4.99 (4.55, 5.65)	4.96 (4.58, 5.57)	5.02 (4.52, 5.83)	0.922
Total cholesterol, median (Q1, Q3)	4.63 (4.12, 5.35)	4.62 (4.16, 5.43)	4.64 (4.05, 5.3)	0.366
Triglyceride, median (Q1, Q3)	1.4 (1.05, 2.01)	1.38 (1.04, 1.97)	1.41 (1.11, 2.07)	0.373
High-density lipoprotein cholesterol, median (Q1, Q3)	1.21 (1.04, 1.4)	1.23 (1.04, 1.44)	1.17 (1.02, 1.36)	0.207
Apolipoprotein A1, median (Q1, Q3)	1.19 (1.08, 1.33)	1.21 (1.07, 1.37)	1.18 (1.08, 1.3)	0.493
Apolipoprotein B, median (Q1, Q3)	0.88 (0.76, 1.04)	0.89 (0.76, 1.03)	0.88 (0.75, 1.05)	0.952
Low-density lipoprotein cholesterol, median (Q1, Q3)	2.83 (2.46, 3.27)	2.83 (2.48, 3.28)	2.83 (2.4, 3.26)	0.562
Potassium, median (Q1, Q3)	3.68 (3.45, 3.89)	3.74 (3.46, 3.92)	3.63 (3.44, 3.81)	0.026
Sodium, median (Q1, Q3)	141 (139, 142)	140 (139, 141)	141 (139, 142)	0.019
Chlorine, median (Q1, Q3)	106 (105, 108)	106 (105, 108)	106 (105, 108)	0.439
Calcium, mean ± SD	2.26 ± 0.1	2.27 ± 0.1	2.25 ± 0.1	0.193
Phosphorus, median (Q1, Q3)	1.12 (1, 1.26)	1.11 (1, 1.24)	1.12 (0.99, 1.28)	0.777
Magnesium, median (Q1, Q3)	0.88 (0.83, 0.91)	0.87 (0.82, 0.9)	0.88 (0.83, 0.92)	0.344
*α*-Hydroxybutyrate dehydrogenase, median (Q1, Q3)	152 (135, 172.25)	147 (131, 169)	154.5 (138, 174.75)	0.004
Lactic dehydrogenase, median (Q1, Q3)	178 (157, 200.25)	172.5 (152, 198)	183 (163, 203)	0.006
Creatine kinase, median (Q1, Q3)	63.5 (48.68, 85.1)	63.5 (49.92, 83.38)	63.55 (47.12, 87.68)	0.721
Creatine kinase isoenzyme, median (Q1, Q3)	12 (9.75, 15)	12 (9, 15)	12 (10, 15)	0.571
Blood urea nitrogen (BUN), median (Q1, Q3)	5.36 (4.48, 6.42)	5.12 (4.3, 5.94)	5.64 (4.7, 6.83)	< 0.001
Creatinine, median (Q1, Q3)	56.7 (50.35, 66.3)	56.95 (50.2, 67.22)	56.4 (50.5, 66.25)	0.98
Uric acid, median (Q1, Q3)	296.25 (248.43, 361.6)	296.6 (250.3, 362.03)	295.45 (245.6, 359.1)	0.928
Bicarbonate, mean ± SD	25.88 ± 2.23	25.83 ± 2.32	25.94 ± 2.13	0.627
*β*2 microglobulin, median (Q1, Q3)	1.63 (1.44, 1.91)	1.53 (1.39, 1.78)	1.72 (1.53, 2.06)	<0.001
Homocysteine determination, median (Q1, Q3)	13.1 (11.38, 16.5)	12.5 (10.9, 16.28)	13.75 (11.9, 16.6)	0.007
Lipoprotein A, median (Q1, Q3)	13.05 (6.68, 28.23)	11.65 (5.62, 26.82)	14.75 (7.53, 29.23)	0.077
Serum cystatin C determination, median (Q1, Q3)	0.64 (0.55, 0.77)	0.6 (0.52, 0.7)	0.67 (0.59, 0.8)	<0.001
Adenosine deaminase, median (Q1, Q3)	9.85 (8.5, 11.9)	9.55 (8.3, 11.7)	10.1 (8.7, 12.2)	0.079
Serum total bile acid, median (Q1, Q3)	3.5 (2.2, 5.7)	3.45 (2.2, 5.77)	3.6 (2.2, 5.5)	0.918
Estimated glomerular filtration rate, median (Q1, Q3)	98.42 (90.7, 104.56)	101.78 (94.04, 108.55)	95.36 (87.9, 100.53)	<0.001
Fibrinogen, median (Q1, Q3)	2.57 (2.25, 2.96)	2.49 (2.26, 2.94)	2.62 (2.24, 2.96)	0.309
Prothrombin time, median (Q1, Q3)	11.2 (10.8, 11.7)	11.2 (10.8, 11.7)	11.2 (10.8, 11.8)	0.693
Thrombin time, median (Q1, Q3)	17.7 (17.1, 18.5)	17.7 (17.1, 18.6)	17.7 (17.1, 18.3)	0.276
Activity, mean ± SD	93.33 ± 8.61	93.52 ± 8.17	93.12 ± 9.08	0.64
International standardized ratio, median (Q1, Q3)	0.97 (0.94, 1.02)	0.97 (0.94, 1.02)	0.97 (0.93, 1.03)	0.666
Activated partial thromboplastin time, mean ± SD	26.08 ± 2.08	26.14 ± 2.12	26.02 ± 2.04	0.563
Fibrinogen degradation products, median (Q1, Q3)	2.5 (2.5, 2.5)	2.5 (2.5, 2.5)	2.5 (2.5, 2.5)	0.103
Antithrombin III, median (Q1, Q3)	87.7 (81.57, 97.73)	87.75 (81.95, 97.18)	87.7 (81.08, 97.85)	0.935
Erythrocyte sedimentation rate, median (Q1,Q3)	10 (6, 17)	9 (5, 15)	12 (6, 18)	0.004
Blood type ABO, *n* (%)				0.766
AB	35 (9)	20 (10)	15 (8)	
A	134 (34)	72 (35)	62 (32)	
B	115 (29)	57 (28)	58 (30)	
O	116 (29)	57 (28)	59 (30)	
Blood type Rh, *n* (%)				1
Negative	2 (0)	1 (0)	1 (1)	
Positive	398 (100)	205 (100)	193 (99)	

## Data Availability

The datasets generated and/or analysed during the current study are not publicly available because of restricted access to our hospital database but are available from the corresponding author upon reasonable request.

## Supplementary Materials

Supplementary Materials Table S1: demographics and clinical characteristics of 172 patients with knee osteoarthritis in the validation cohort. Figure S1: dynamic nomogram prediction of osteoarthritis severity in patients with knee osteoarthritis.
