# Supplementary material for: A Simple Nomogram for Predicting Osteoarthritis Severity in Patients with Knee Osteoarthritis
Source: Comput Math Methods Med. 2022 Sep 2;2022:3605369. doi: 10.1155/2022/3605369 (PMC9462991; doi:10.1155/2022/3605369)
Supplement: Supplementary Materials — Table S1: demographics and clinical characteristics of 172 patients with knee osteoarthritis in the validation cohort. Figure S1: dynamic nomogram prediction of osteoarthritis severity in patients with knee osteoarthritis. [file 3605369.f1.doc]

**additional file**

Table S1. Demographics and clinical characteristics of 172 patients with knee osteoarthritis in the validation cohort.

| Variables | Total (n = 172) | KL 1-3(n = 67) | K-L 4(n = 105) | p |
| --- | --- | --- | --- | --- |
| Sex, n (%) |  |  |  | 0.652 |
| Female | 129 (75) | 52 (78) | 77 (73) |  |
| Male | 43 (25) | 15 (22) | 28 (27) |  |
| Age, Mean ± SD | 62.41 ± 6.36 | 59.54 ± 6.56 | 64.24 ± 5.51 | < 0.001 |
| Height, Median (Q1,Q3) | 160 (157, 165) | 160 (157, 165.5) | 160 (157, 165) | 0.382 |
| Weight, Median (Q1,Q3) | 70 (64.75, 80) | 70 (64.5, 80) | 70 (65, 80) | 0.499 |
| Physical illnesses  , n (%) |  |  |  | 0.074 |
| No | 52 (30) | 26 (39) | 26 (25) |  |
| Yes | 120 (70) | 41 (61) | 79 (75) |  |
| Temperature,Median (Q1,Q3) | 36.5 (36.5, 36.6) | 36.5 (36.45, 36.6) | 36.5 (36.5, 36.6) | 0.709 |
| Pulse, Median (Q1,Q3) | 80 (75, 88) | 76 (72, 80) | 80 (76, 88) | 0.003 |
| Breathing rate, Median (Q1,Q3) | 20 (18, 22) | 19 (18, 20) | 20 (19, 22) | 0.004 |
| Systolic pressure, Mean ± SD | 145.94 ± 18.23 | 142.3 ± 17.14 | 148.27 ± 18.61 | 0.033 |
| Diastolic pressure  , Mean ± SD | 91.09 ± 10.9 | 90.55 ± 11 | 91.43 ± 10.88 | 0.61 |
| C-reactive protein, Median (Q1,Q3) | 1.82 (1.29, 3.45) | 1.67 (1.19, 2.95) | 2.12 (1.35, 4.06) | 0.104 |
| White blood cell count, Median (Q1,Q3) | 5.26 (4.59, 6.15) | 5.03 (4.43, 5.97) | 5.39 (4.73, 6.19) | 0.095 |
| Red blood cell count, Mean ± SD | 4.3 ± 0.46 | 4.29 ± 0.44 | 4.31 ± 0.47 | 0.716 |
| Haemoglobin, Median (Q1,Q3) | 131.85 (122.68, 139.85) | 131.2 (123.8, 141.6) | 132 (121.6, 139) | 0.652 |
| Haematocrit, Mean ± SD | 39.13 ± 4.25 | 39.31 ± 3.78 | 39.02 ± 4.54 | 0.647 |
| Platelet count, Median (Q1,Q3) | 233.3 (202.42, 271.17) | 229 (201.4, 267.35) | 236 (202.8, 282.1) | 0.211 |
| Neutrophil ratio, Mean ± SD | 57.94 ± 8.43 | 57.14 ± 8.92 | 58.46 ± 8.1 | 0.331 |
| Lymphocyte percentage, Mean ± SD | 31.77 ± 7.55 | 32.59 ± 7.75 | 31.24 ± 7.4 | 0.259 |
| Monocyte percentage, Mean ± SD | 7.38 ± 1.86 | 7.33 ± 1.92 | 7.42 ± 1.83 | 0.757 |
| Percentage of eosinophils, Median (Q1,Q3) | 1.89 (1.2, 3.09) | 1.96 (1.25, 3.09) | 1.88 (1.1, 3.2) | 0.873 |
| Percentage of basophils, Median (Q1,Q3) | 0.56 (0.4, 0.75) | 0.51 (0.4, 0.76) | 0.57 (0.4, 0.75) | 0.699 |
| Absolute value of neutrophils, Median (Q1,Q3) | 3.04 (2.39, 3.86) | 2.78 (2.24, 3.58) | 3.07 (2.58, 3.88) | 0.13 |
| Absolute value of lymphocytes, Median (Q1,Q3) | 1.68 (1.37, 1.92) | 1.67 (1.37, 1.9) | 1.69 (1.36, 1.94) | 0.777 |
| Absolute value of monocytes, Median (Q1,Q3) | 0.38 (0.31, 0.48) | 0.37 (0.3, 0.46) | 0.39 (0.33, 0.48) | 0.207 |
| Absolute value of eosinophils, Median (Q1,Q3) | 0.1 (0.07, 0.16) | 0.1 (0.07, 0.14) | 0.1 (0.07, 0.18) | 0.744 |
| Absolute value of basophils, Median (Q1,Q3) | 0.03 (0.02, 0.04) | 0.03 (0.02, 0.04) | 0.03 (0.02, 0.04) | 0.517 |
| Average volume of red blood cells, Median (Q1,Q3) | 90.85 (88.68, 93.76) | 91.24 (89.28, 93.8) | 90.42 (88.4, 93.54) | 0.13 |
| Average haemoglobin content, Median (Q1,Q3) | 30.4 (29.51, 31.52) | 30.61 (30.16, 31.6) | 30.16 (29.23, 31.39) | 0.031 |
| Mean corpuscular haemoglobin concentration(MCHC), Median (Q1,Q3) | 334.5 (329.88, 339.4) | 335.5 (331.75, 341.75) | 333.7 (328, 339) | 0.024 |
| Coefficient of the variation of red blood cell distribution width, Median (Q1,Q3) | 13.04 (12.61, 13.49) | 12.83 (12.56, 13.21) | 13.18 (12.66, 13.64) | 0.032 |
| Average volume of platelets, Median (Q1,Q3) | 8.74 (8.09, 9.71) | 8.7 (8.07, 9.8) | 8.77 (8.09, 9.7) | 0.91 |
| Distribution width of platelets, Median (Q1,Q3) | 16.52 (15.89, 16.8) | 16.6 (15.99, 16.84) | 16.43 (15.84, 16.79) | 0.456 |
| Thrombocytocrit, Median (Q1,Q3) | 0.21 (0.18, 0.25) | 0.2 (0.18, 0.22) | 0.21 (0.18, 0.26) | 0.329 |
| Total protein, Mean ± SD | 68.97 ± 5.8 | 68.21 ± 5.89 | 69.45 ± 5.72 | 0.175 |
| Albumin, Mean ± SD | 42.21 ± 3.35 | 42.25 ± 3.28 | 42.19 ± 3.41 | 0.917 |
| Total bilirubin, Median (Q1,Q3) | 13.06 (10.66, 18.08) | 13 (10.71, 15.85) | 13.34 (10.6, 18.83) | 0.582 |
| Prealbumin, Mean ± SD | 240.84 ± 40.93 | 248.5 ± 39.71 | 235.96 ± 41.14 | 0.048 |
| Alanine aminotransferase, Median (Q1,Q3) | 17.5 (13, 23.25) | 18 (14, 25) | 17 (12, 22) | 0.135 |
| Aspartate aminotransferase, Median (Q1,Q3) | 18 (15.75, 21.25) | 18 (16, 22.5) | 18 (15, 21) | 0.439 |
| Gamma glutamyltransferase, Median (Q1,Q3) | 19 (15, 27) | 22 (16, 30) | 19 (14, 24) | 0.002 |
| Direct bilirubin, Median (Q1,Q3) | 3.92 (2.95, 5.08) | 3.62 (2.95, 4.58) | 3.97 (2.99, 5.29) | 0.236 |
| Alkaline phosphatase, Median (Q1,Q3) | 79.5 (69, 93) | 78 (65.5, 86.5) | 80 (70, 97) | 0.098 |
| Blood glucose, Median (Q1,Q3) | 5.42 (5.01, 6.03) | 5.29 (4.96, 5.8) | 5.5 (5.06, 6.21) | 0.055 |
| Total cholesterol, Mean ± SD | 5.08 ± 0.96 | 5.11 ± 0.98 | 5.06 ± 0.94 | 0.714 |
| Triglyceride, Median (Q1,Q3) | 1.42 (1.11, 1.94) | 1.44 (1.11, 1.88) | 1.4 (1.11, 1.97) | 0.745 |
| High-density lipoprotein cholesterol, Mean ± SD | 1.28 ± 0.23 | 1.25 ± 0.21 | 1.3 ± 0.24 | 0.192 |
| Apolipoprotein A1, Mean ± SD | 1.33 ± 0.19 | 1.31 ± 0.18 | 1.34 ± 0.19 | 0.298 |
| Apolipoprotein B, Mean ± SD | 0.99 ± 0.23 | 1.01 ± 0.24 | 0.98 ± 0.22 | 0.4 |
| Low-density lipoprotein cholesterol, Mean ± SD | 2.97 ± 0.66 | 3.01 ± 0.69 | 2.94 ± 0.65 | 0.515 |
| Potassium, Median (Q1,Q3) | 4 (3.81, 4.21) | 4 (3.83, 4.18) | 3.98 (3.77, 4.26) | 0.667 |
| Sodium, Median (Q1,Q3) | 141.2 (139.94, 142.49) | 141.22 (140.14, 142.5) | 141.17 (139.78, 142.3) | 0.726 |
| Chlorine, Median (Q1,Q3) | 106.06 (104.25, 107.8) | 106.71 (104.5, 108.32) | 105.91 (104.21, 107.64) | 0.152 |
| Calcium, Mean ± SD | 2.29 ± 0.1 | 2.29 ± 0.11 | 2.29 ± 0.1 | 0.849 |
| Phosphorus, Mean ± SD | 1.23 ± 0.15 | 1.23 ± 0.15 | 1.22 ± 0.16 | 0.571 |
| Magnesium, Median (Q1,Q3) | 0.93 (0.88, 0.98) | 0.94 (0.88, 0.97) | 0.93 (0.88, 0.98) | 0.961 |
| α-hydroxybutyrate  dehydrogenase  , Median (Q1,Q3) | 147.3 (134.71, 163.55) | 144.31 (132.4, 167.99) | 147.6 (136.87, 160.65) | 0.632 |
| href="javascript:;" Lactic [dehydrogenase](../../../../H:/zhangqingzhu/投稿/2修/javascript:%3B), Median (Q1,Q3) | 176.08 (162.56, 198.17) | 175.22 (160.56, 206.5) | 176.69 (163.95, 194.45) | 0.926 |
| [Creatine](../../../../H:/zhangqingzhu/投稿/2修/javascript:%3B) [kinase](../../../../H:/zhangqingzhu/投稿/2修/javascript:%3B), Median (Q1,Q3) | 74 (55, 98.72) | 79 (55.05, 98.1) | 71.6 (55, 99.1) | 0.623 |
| [Creatine](../../../../H:/zhangqingzhu/投稿/2修/javascript:%3B) [Kinase](../../../../H:/zhangqingzhu/投稿/2修/javascript:%3B) [Isoenzyme](../../../../H:/zhangqingzhu/投稿/2修/javascript:%3B), Median (Q1,Q3) | 12.71 (10.13, 15.92) | 12.68 (9.38, 15.68) | 12.86 (10.36, 16.01) | 0.583 |
| Blood urea nitrogen(BUN), Median (Q1,Q3) | 5.14 (4.5, 6.25) | 4.91 (4.31, 6.08) | 5.37 (4.59, 6.26) | 0.14 |
| Creatinine, Median (Q1,Q3) | 60.38 (52.97, 69.83) | 59.72 (50.57, 68.33) | 60.43 (54.24, 71.02) | 0.422 |
| Uric acid, Mean ± SD | 301.44 ± 66.74 | 301.97 ± 72.28 | 301.1 ± 63.32 | 0.936 |
| Bicarbonate, Median (Q1,Q3) | 23.29 (22.08, 25.28) | 23.09 (21.93, 25.32) | 23.41 (22.16, 25.25) | 0.418 |
| Homocysteine determination, Median (Q1,Q3) | 11 (9.4, 13.7) | 10.2 (8.7, 12.2) | 11.5 (9.8, 14.2) | 0.008 |
| Lipoprotein A, Median (Q1,Q3) | 180 (88.88, 387.93) | 229.9 (93.55, 411.9) | 170.6 (84.3, 372.9) | 0.59 |
| Serum total bile acid, Median (Q1,Q3) | 3.3 (2.1, 5.73) | 3 (1.95, 5.15) | 3.5 (2.2, 6.2) | 0.127 |
| Fibrinogen, Median (Q1,Q3) | 3.08 (2.81, 3.42) | 3.06 (2.79, 3.45) | 3.09 (2.83, 3.39) | 0.863 |
| Prothrombin time, Median (Q1,Q3) | 11.3 (11, 11.83) | 11.3 (10.95, 11.7) | 11.3 (11, 11.9) | 0.95 |
| Thrombin time, Mean ± SD | 14.22 ± 0.97 | 14.2 ± 1.1 | 14.23 ± 0.88 | 0.853 |
| Activity, Mean ± SD | 96.24 ± 8.99 | 96.21 ± 7.54 | 96.26 ± 9.84 | 0.971 |
| International standardized ratio, Median (Q1,Q3) | 1.02 (0.99, 1.05) | 1.02 (0.98, 1.05) | 1.02 (0.99, 1.06) | 0.901 |
| Activated partial thromboplastin time, Mean ± SD | 31.5 ± 2.74 | 31.96 ± 2.61 | 31.21 ± 2.8 | 0.078 |
| Fibrinogen degradation products, Median (Q1,Q3) | 1.03 (0.75, 1.5) | 0.83 (0.64, 1.25) | 1.12 (0.81, 1.55) | 0.005 |
| Antithrombin III, Median (Q1,Q3) | 92 (85, 100.25) | 93 (85, 101) | 91 (85, 100) | 0.623 |
| Erythrocyte Sedimentation rate, Median (Q1,Q3) | 8 (5, 13) | 8 (6, 13) | 8 (5, 13) | 0.512 |
| Blood type ABO, n (%) |  |  |  | 0.462 |
| AB | 13 (8) | 4 (6) | 9 (9) |  |
| A | 52 (30) | 19 (28) | 33 (31) |  |
| B | 57 (33) | 20 (30) | 37 (35) |  |
| O | 50 (29) | 24 (36) | 26 (25) |  |
| Blood type Rh, n (%) |  |  |  | 1 |
| negative | 1 (1) | 0 (0) | 1 (1) |  |
| positive | 171 (99) | 67 (100) | 104 (99) |  |


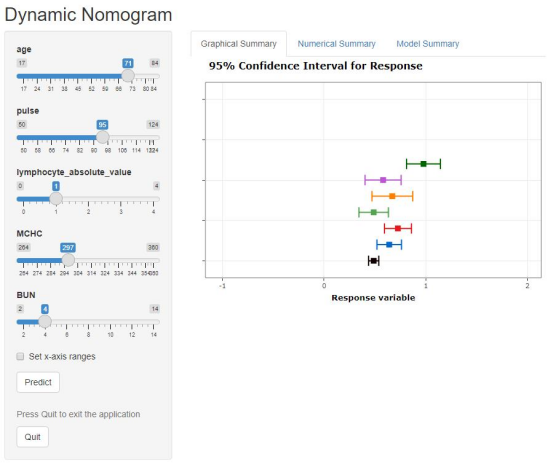


Figure S1. Dynamic nomogram prediction of osteoarthritis severity in patients with knee osteoarthritis.
